# Advances in arterial spin labelling MRI methods for measuring perfusion and collateral flow

**DOI:** 10.1177/0271678X17713434

**Published:** 2017-06-09

**Authors:** Matthias JP van Osch, Wouter M Teeuwisse, Zhensen Chen, Yuriko Suzuki, Michael Helle, Sophie Schmid

**Affiliations:** 1 Department of Radiology, Leiden University Medical Center, Leiden, The Netherlands; 2 Leiden Institute of Brain and Cognition, Leiden University, Leiden, The Netherlands; 3 Department of Biomedical Engineering, Tsinghua University, Beijing, China; 4 Philips GmbH Innovative Technologies, Research Laboratories, Hamburg, Germany

**Keywords:** Arterial spin labelling, cerebral blood flow, cerebral haemodynamics, magnetic resonance, perfusion weighted MRI

## Abstract

With the publication in 2015 of the consensus statement by the perfusion study group of the International Society for Magnetic Resonance in Medicine (ISMRM) and the EU-COST action ‘ASL in dementia’ on the implementation of arterial spin labelling MRI (ASL) in a clinical setting, the development of ASL can be considered to have become mature and ready for clinical prime-time. In this review article new developments and remaining issues will be discussed, especially focusing on quantification of ASL as well as on new technological developments of ASL for perfusion imaging and flow territory mapping. Uncertainty of the achieved labelling efficiency in pseudo-continuous ASL (pCASL) as well as the presence of arterial transit time artefacts, can be considered the main remaining challenges for the use of quantitative cerebral blood flow (CBF) values. New developments in ASL centre around time-efficient acquisition of dynamic ASL-images by means of time-encoded pCASL and diversification of information content, for example by combined 4D-angiography with perfusion imaging. Current vessel-encoded and super-selective pCASL-methodology have developed into easily applied flow-territory mapping methods providing relevant clinical information with highly similar information content as digital subtraction angiography (DSA), the current clinical standard. Both approaches seem therefore to be ready for clinical use.

## Introduction

The brain lacks almost any form of energy storage and therefore all energy needs to be transported into the brain by means of cerebral perfusion.^1^ Even short interruptions of the blood supply can lead to a dramatic loss of neurons (1 million/min), synapses (14 billion/min) and myelinated fibres (12 km/min).^2^ Furthermore, the seminal work of Astrup in 1981 appeared to indicate well-defined thresholds for cerebral blood flow (CBF)-values for tissue destined for infarction (i.e. core of an infarct) or for tissue in which energy failure occurs, but the tissue is still salvageable (penumbra).^3^ These observations resulted in a quest for imaging approaches to be able to detect *hypo*perfusion. At the same time, the amount of blood flow to (brain) tumours was found to be an important marker for the grade of the tumour with higher perfusion found to be associated with higher tumour-grade.^4–6^ Additionally, the observation of functional hyperemia, i.e. the disproportional larger increase in blood flow following neuronal activation, made it necessary to develop perfusion magnetic resonance imaging (MRI) methods that could detect *hyper*perfusion.^7–9^ These three main application areas (stroke, cancer and functional imaging) have been the driving forces of perfusion MRI over the last three decades and motivated the MRI community to develop non-invasive, accurate and quantitative cerebral perfusion approaches.^10–14^ The application of cerebral perfusion as research tool has gained even more importance in the last few years by the recognition of the concept of the neurovascular unit, as well as proof from large patient studies that vascular factors are important risk factors in many neurodegenerative diseases (fourth application area).^15,16^ For example, a recent publication based upon the data from the Alzheimer’s Disease Neuroimaging Initiative (ADNI) found that MR perfusion imaging should be considered the earliest as well as most predictive bio-marker for Alzheimer disease.^17^ Moreover, perfusion in the posterior cingulate cortex has been shown to be a good predictor of deteriorating cognitive functioning.^18^ The fifth important application area of perfusion MRI encompasses patients with large vessel disease, who show adaptations of the haemodynamic involvement of their vascular architecture, most notably via collateral blood flow via the Circle of Willis.^19–21^

Perfusion MRI can be performed by monitoring the first-passage of a bolus of contrast agent (dynamic susceptibility contrast (DSC) MRI)^22–25^ or completely non-invasive by magnetically labelling the inflow of arterial blood (arterial spin labelling (ASL) MRI)^12,26^ or by exploiting motion sensitizing gradients (intra-voxel incoherent motion (IVIM)).^13^ All these techniques were first proposed in the late 80 s and early 90 s of the last century, but many further improvements in MRI hardware, sequence optimization, post-processing and interpretation were needed before these approaches could be considered mature. In all these years, many of these basic approaches have been extended to obtain more information from the cerebral microvasculature than just the blood flow. Especially, for ASL many new developments can be observed in the last few years and new concepts are continuously emerging. In this review article, these recent developments in ASL-imaging methodology for perfusion imaging and collateral blood flow will be discussed and avenues of future research will be identified. The starting point of this review will be the state-of-the-art approach for measuring CBF as published in the consensus paper by the perfusion study group of the International Society for Magnetic Resonance in Medicine (ISMRM) and the EU-COST action ‘ASL in dementia’.^27^ Subsequently, the focus will shift towards new approaches that solve remaining issues as identified in the consensus paper or that can obtain information from the cerebral haemodynamics beyond CBF.

## Current state-of-the-art of ASL

ASL-perfusion MRI is based upon the use of blood as an endogenous tracer by employing spatially selective labelling of the inflowing blood that inverts its longitudinal magnetization. After a typical delay-time (post-labelling delay, PLD) of approximately 2 s a fast readout imaging module is employed to map the brain magnetization. After subtraction of such a ‘label’ image from a ‘control’ scan that is identical to the label condition except for the absence of inversion of inflowing blood, an image of the labelled blood that has reached the brain tissue is obtained. This subtraction image can be quantified to obtain a CBF-map when the temporal width of the bolus of labelled spins is taken into account and by correcting for the decay of label due to longitudinal relaxation (T_1_).

With the publications in 2014 (early view publication; printed version available early 2015) of the consensus statement by the perfusion study group of the ISMRM and the EU-COST action ‘ASL in dementia’ on the implementation of ASL in a clinical setting, the development of ASL can be considered to have become mature and ready for clinical prime-time.^27^ In this consensus statement, pseudo-continuous arterial spin labelling (pCASL) has been adopted as the work-horse technique for perfusion imaging in clinical application. This labelling approach was recommended to be combined with background suppression, sufficiently long delay times (>1500 ms depending on the age and condition of the subject, e.g. 1800 ms in healthy subjects younger than 70 years and 2000 ms in healthy subjects older than 70 years) and a segmented 3D readout. Sequences closely adhering to the consensus settings are now available as product or ‘work-in-progress’ packages on scanners of the three leading MRI vendors. Comparison studies in normal subjects showed that pCASL CBF-maps acquired with sequences closely matching the consensus settings, provide similar qualitative and quantitative maps as gold standard [^15^O]H_2_O Positron Emission Tomography images (PET).^28–30^ Moreover, pCASL CBF increases upon a hypercapnia challenge were found to be equal in normal volunteers as those measured with PET.^28^ Also in patients with Alzheimer’s Disease, similar regions of hypometabolism were identified by pCASL as by FDG-PET.^31^ The combination of improved image quality, the consensus statement, the improved availability of the sequence and the validation studies has led to a sharp increase in the use of ASL in (clinical) research. This is evidenced by the number of publications: in 2000, approximately 25 papers were published with ‘Arterial spin label(l)ing’ in the topic or title, which increased to 80 publications in 2005, 145 in 2010 and 275 publications in 2015 (source: Web of Science, Thomas Reuters, Toronto, Canada).

## Post-consensus era: Remaining quantification issues

**Figure 1.**
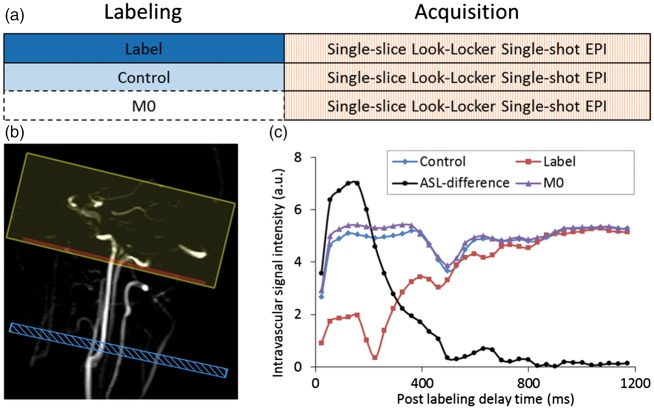
Overview of a direct measurement of the labelling efficiency of pCASL with the schematic representation of the acquisition protocol in (a), the planning in (b) and the observed signals in (c). The passage of labelled spins is measured in an imaging plane superior from the labelling slab. Readout is performed with a Look-Locker readout with 90° excitation pulses assuming complete refreshment of spins within the imaging plane between two excitation pulses. Similarly, the passage of spins that have either experienced the control condition of the pCASL-labelling or which did not experience any RF (‘M0’) are measured. By correcting the ASL-signal (control-label) for T1-decay and by normalizing with respect to M0, the labelling efficiency can be calculated. Note that the first points of the label-curve represent negative magnetization, which has been made positive by the modulus operator; this has been taken into account in the curve showing the ASL-difference signal. For details, see Chen et al.^37^ Blue box indicates labelling plane, the red box the imaging slice and the yellow box a saturation volume to suppress venous signal.

**Figure 2.**
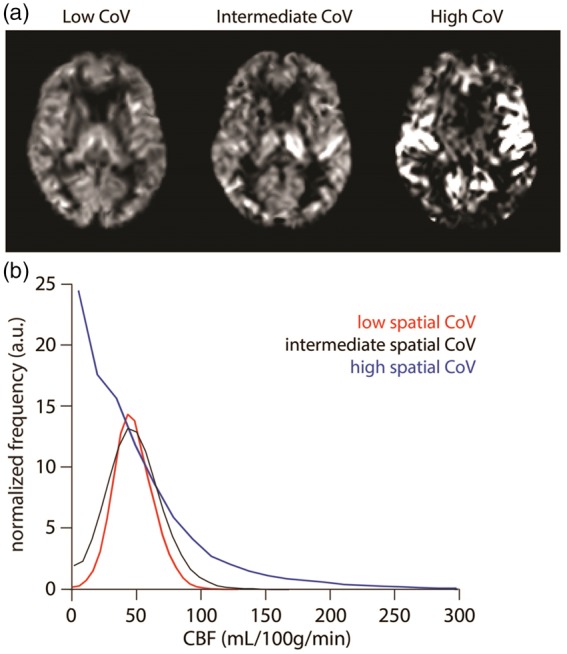
Cerebral blood flow (CBF) images (a) and histograms (b) are shown for three selected participants from a larger clinical study with, respectively, the lowest (39.3%), intermediate (54.0%) and highest (113.6%) spatial coefficient of variation (CoV). Histograms proof that the subject with highest CoV has many voxels with almost zero CBF, but also a large tail of voxels with high CBF; both findings point to increased arterial transit time. For the intermediate and low CoV, the histograms become more-and-more symmetric with less-and-less outlier voxels with high CBF-values. Image courtesy: Henk-Jan Mutsaerts (Brain Sciences Research Program, Sunnybrook Research Institute, Toronto, Canada). Image reproduced with permission from Mutsaerts et al.^62^

The consensus statement included a deliberately simplified model for quantification of pCASL-data to limit the amount of model-parameters and to stimulate uniformity between different centres and researchers. Three main quantification issues can be considered to be the dominating sources of errors in quantification when using this simplified model, especially when applied to patients or elderly subjects:

1. Uncertainty in labelling efficiency The quantification formula of the consensus statement advices to employ a constant labelling efficiency of 85% as taken from simulation studies.^32^ It is, however, known that the labelling efficiency of pCASL can vary significantly as a function of blood velocity and due to off-resonance effects within the labelling plane.^32,33^ Because labelling efficiency directly affects the quantitative numbers of the pCASL-experiment, the uncertainty in labelling efficiency might limit the use of quantitative numbers in individual patients. Four different approaches have recently been proposed to mitigate this issue:
  - Adaptation of the phase of the PCASL RF-pulses The labelling of pCASL is based upon a flow induced phase change over a long train of short RF-pulses, which results in a pseudo-adiabatic inversion of the blood magnetization. Both off-resonance effects as well as different blood velocities can influence the coherent phase built-up and can therefore decrease labelling efficiency. For balanced pCASL, the only difference between the label and the control condition is a π phase difference between every two consecutive RF-pulses. This clearly shows that additional phase accrual caused by e.g. off-resonance can seriously affect the labelling efficiency. By deliberately adding additional phase accruals to the train of RF-pulses, the optimal labelling efficiency can be experimentally achieved (multi-phase (MP-)pCASL).^33,34^ An alternative option is to measure the off-resonance at the vessel locations by means of a field map and to translate the local off-resonance to the optimal phase accrual.^35^
  - Minimizing the influence of blood velocity and off-resonance effects Zhao et al. were recently able to achieve significant higher robustness for pCASL-labelling, as compared to the recommended settings,^27^ by careful optimization of the strength of the mean gradient played out in the blood flow direction, as well as by optimally choosing the strength of the slice-selection gradient under the RF-pulses.^36^ More specific, this was achieved by using unbalanced pCASL in combination with the highest achievable B_1_ and a relatively small mean, slice-selective pCASL-gradient. With the optimized settings the variance in labelling efficiency was found to be a factor 2.5 smaller than with the settings from the consensus paper.
  - Direct measurement The labelling efficiency can also be measured by a separate scan, so that a true estimate of this parameter can be included in the quantification process (see Figure 1).^37^ Importantly, this can not only be measured for each individual or scan, but also for each artery, thereby improving the interpretation of CBF-maps both qualitatively as well as quantitatively. Measurement of the labelling efficiency can be achieved by acquiring a separate scan that monitors the passage of labelled spins in an imaging slice above the labelling plane, see the red plane in Figure 1. Although this approach will miss the first labelled spins (they will already have passed the imaging plane before the start of the readout), it will capture the outflow of the last labelled spins. From the temporal shape of the outflow curve, the transport time from the labelling plane can be inferred, which is necessary to correct for the loss of label due to longitudinal relaxation. This approach can measure the labelling efficiency in the main brain feeding arteries (internal carotid- and vertebral arteries) with reasonable precision and robustness. Since the labelling efficiency can be measured in the different arteries, it will enable to discriminate asymmetrical perfusion maps due to true perfusion differences from artefactual asymmetries caused by different labelling efficiencies of the different brain-feeding arteries.
  - Indirect measurement The earliest proposed method to correct for the influence of sub-optimal labelling efficiency on CBF-quantification of pCASL-scans involves an additional quantitative flow measurement by means of phase contrast MR-angiography (PC-MRA) and a brain tissue volume estimation obtained by segmentation of a 3D-T_1_ anatomical scan.^38^ Since the average brain perfusion from the pCASL-sequence should be equal to the total blood flow to the brain as measured by PC-MRA divided by the brain volume, an estimate of the average labelling efficiency can be obtained. Drawback of this approach is that only the average labelling efficiency can be obtained, i.e. not the labelling efficiency of individual brain feeding arteries. Although one could envision that a similar approach could be performed by calculating the volume of flow territories of the involved arteries based on the combination of a flow territory scan and a 3D-T_1_ scan.
2. Differences in T_1_ of arterial blood Since the labelling process is based on inversion of the blood magnetization, the amount of label created will decay due to T_1_-relaxation. To be able to correct for this loss, it is necessary to assume a value for the longitudinal relaxation time of the labelled spins within the quantification process. Since in an ASL-experiment the label resides most of the time in blood, it was proposed in the consensus paper to assume that the label resides the whole experiment in blood and therefore that the loss of label is governed by the T_1_ of blood. Previously, it has been established that this is a reasonable approach when the time of exchange from intra- to extravascular compartment is unknown, by comparing quantitatively this approach with a two compartment model.^39^ The main quantification issue arises from the well-known fact that the T_1_ of blood depends both on the haematocrit and other blood constituents.^40–42^ Moreover, many diseases may influence these factors, which can result in slower or faster decay of the ASL-label, and therefore alter the quantitative values of the CBF-map independent of the underlying perfusion.^43–46^ This becomes especially a problem when comparing a patient with a control group, when even only subtle differences in e.g. haematocrit could already lead to significant differences in CBF as measured by ASL when assuming equal T_1_ of blood for both groups. Finally, it should be realized that also gender differences in haematocrit have an effect on CBF-values as obtained by ASL. Since women have on average a lower haematocrit than men, women have on average a longer T_1,blood_ resulting in an overestimation of CBF-values when not corrected for.^47^ When comparing the ASL-signal with T_1_ of blood of an average female subject (T_1_ = 1681 ms) to that of a typical male (T_1_ = 1618 ms) at 3 T, the signal will be 6% higher for the female when all other factors are equal (estimation based upon the ASL-settings and quantification formula of the consensus paper).^27,47^ To account for these quantification errors, the T_1_ of blood can be measured by a quick scan as proposed by Varela et al.^46^ Drawback of that approach was that it measured the T_1_ in venous blood, whereas for ASL-quantification, the T_1_ of arterial blood is more relevant. Recently, Li et al. have published an extension of the Varela approach, which makes it possible to measure the *arterial* blood T_1_ within a similar short scan-time.^48^
3. Variation in transport times The fundamental compromise for any ASL-sequence arises from balancing long enough PLDs to guarantee that all labelled spins have arrived in the tissue compartment ensuring accurate quantification, with short enough PLDs to ensure detection of sufficient ASL-signal for a robust measurement before all label has decayed to the noise level due to longitudinal relaxation.^10,49^ This is for example evident in the deep white matter, where little or no ASL-signal can be detected with the consensus settings due to slow arrival of labelled blood.^50,51^ Increasing the PLD and the number of averages does, however, not result in sufficient ASL-signal within the deep white matter to obtain a reliable measurement of the CBF.^52^ The influence of different arterial transit times (ATT) on quantitative CBF-values can be complicated, since it affects quantification in two ways. First, it can lead to underestimation of CBF, because not all labelled spins have arrived in their destination voxel. But it could also result in an overestimation of CBF, since label decays faster in tissue (T_1_ of gray matter is approximately 1200 ms at 3 T) than in blood (T_1_ of blood is approximately 1650 ms).^41^ The last effect results in e.g. an apparent 7% increase in CBF when the arrival-time would increase from 900 till 1200 ms (i.e. in both cases, it is assumed that all label has arrived in the tissue compartment; calculated with a 1800 ms labelling duration and 1800 ms PLD). When the label spends more time in blood due to prolonged arterial transit times, it will have decayed to a lesser extent than when it quickly moves into the extravascular compartment. Whereas multi-timepoint ASL by means of a Look-Locker readout does allow the detection of delayed arrival of label, this comes at the expense of a smaller coverage and a *lower* SNR at the longer PLDs due to the fact that earlier readout pulses will have effectively decreased the amount of signal available for later PLDs.^53,54^ Alternatively, multiple pCASL-scans can be obtained, each with a different PLD and/or labelling duration.^55,56^ Whenever the total acquisition time is kept constant, this approach will automatically limit the number of averages that can be obtained. However, it can still be argued that availability of information on arrival-time as obtained from multi-PLD data outweighs the disadvantage of lower SNR during the actual perfusion phase due to lower number of averages for two reasons. First of all, arrival time itself can be an important parameter providing strong proof of a haemodynamic compromised status. This echoes, for example, the findings by DSC-MRI in acute stroke trials in which the timing information is often found to be more predictive than changes in CBF.^57–59^ Second, the measurement of the arrival time allows to improve the absolute quantification of CBF, which may increase its clinical value. A more efficient way to acquire multi-timepoint ASL, time encoded pCASL, is a relatively new approach suffers less from an SNR-penalty and will be discussed in a separate paragraph.^60^ Besides these acquisition options to measure arterial transit times, another strategy would be to estimate or even to quantify such effects based only on the data of a single PLD perfusion map. Whereas an ASL-acquisition with and without vascular crushers might enable the identification of intravascular signal,^61^ visual inspection of non-crushed CBF-maps is also quite sensitive in detecting intravascular label based upon the punctuate pattern of hyperintense signal. Mutsaerts et al. have automated this approach by calculating the spatial covariance over a gray matter mask.^62^ This covariance measure has the potential to act as a surrogate marker of arterial transit time effects (see Figure 2). Finally, the already mentioned approach of visual recognition of intravascular signal by a human observer should be considered a separate approach on itself and it is especially important to take this finding into account during the diagnosis and/or interpretation process of ASL-images.^27,63^ In this sense, it is important that radiologists and clinicians report ASL-scans as ‘ASL-scans’ instead of ‘perfusion scans’, i.e. an artefact such as intravascular signal should be considered important information on the haemodynamic status of the patient and not just a reason to disregard the whole scan because of severe artefacts and/or acquisition issues.

## Time-encoded ASL

**Figure 3.**
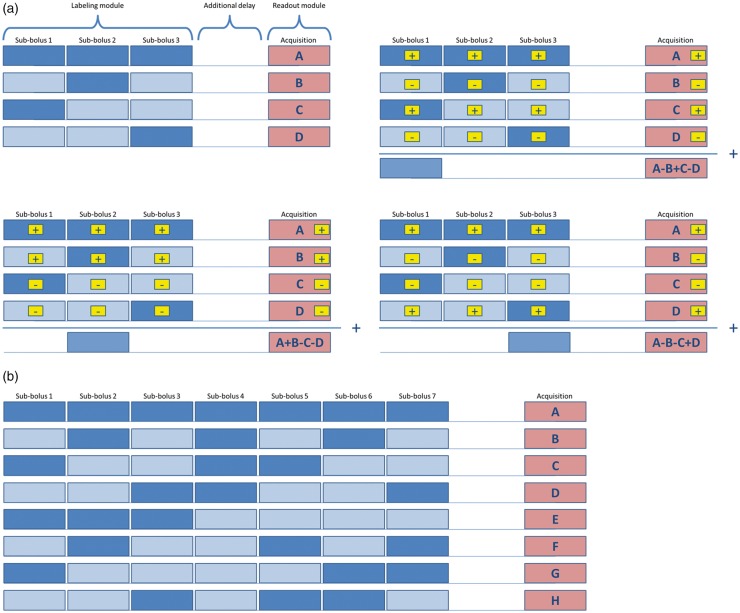
Schemes of Hadamard-4 (a) and Hadamard-8 (b) matrices for application in time-encoded ASL. For Hadamard-4 not only the encoding matrix is shown but also the three decoding options are presented that are employed for calculation of the individual sub-boli images. Summation (or subtraction) of images is indicated in the yellow boxes. Below the decoding matrix, the traditional (non time-encoded) ASL-scan is indicated, which will provide the same image with the same SNR as the decoded sub-bolus image (when similar total scan time is employed). Dark blue boxes indicate label condition of the pCASL-labelling; light blue boxes with dark blue wavy patterns indicate control condition of the pCASL-labelling; dark blue boxes with light blue wavy patterns indicate the result of the subtraction of control minus label condition, i.e. the ASL-signal. Pink boxes indicate imaging.

Günther proposed in 2009 the concept of temporal encoding in a Hadamard fashion of a continuous ASL-scan as a highly time-efficient approach to obtain multi-PLD data.^60^ The basic idea of this approach lies in the observation that it is necessary to average ASL data over many acquisitions to obtain sufficient SNR. Time-encoded ASL (te-ASL) relies on encoding slightly different information content into each repetition, as opposed to repeating the same sequence over-and-over again. Whereas traditionally the pCASL-preparation module has only a single condition, i.e. labelling-only or control-only, in te-pCASL, a Hadamard matrix of rank H is applied to (a) divide the preparation module into (H-1) blocks, effectively splitting the labelling period into sub-boli, (b) to determine for each of H repeated acquisitions the condition (label or control) for each block according to an Hadamard matrix and (c) to decode the acquired images in post-processing. This approach renders (H-1) ASL maps that are similar to traditional ASL-scans with the labelling duration equal to the block duration and a PLD equal to the time-difference between the end of a block and the start of image acquisition. This principle is illustrated in Figure 3 for a Hadamard-4 matrix, whereas also the encoding scheme of a Hadamard-8 te-ASL scan is shown. Since all images are employed and are weighted similarly during the decoding process, i.e. every image is either added or subtracted during decoding, the SNR of this approach is similar to the SNR of a traditional single PLD ASL-scan of equal scan duration. This implies that while the reconstructed ASL-scan of the first sub-bolus has the same SNR as the corresponding traditional ASL-scan, the other reconstructed sub-boli provide information ‘for free’. This poses therefore a very attractive approach when temporal information on the inflow of labelled spins is needed or when one wants to track properties of labelled spins while flowing through the vascular tree. Before show-casing some applications of time-encoded ASL, some pitfalls of this approach are discussed.

### Pitfall 1: Low SNR of images from early labelled sub-boli

**Figure 4.**
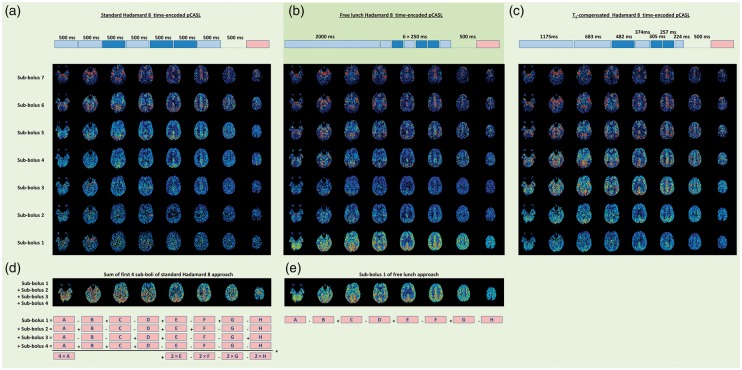
The result of three Hadamard time-encoded, background suppressed pCASL-scans of 5.5 min each (scans acquired on a 3 T MRI scanner (Ingenia, Philips, The Netherlands) on a 43-year-old volunteer who provided informed consent under an approved IRB-protocol; scanner software was locally adapted to allow time-encoded ASL); all sub-boli images have been corrected for T1-decay of label based upon a T1 of 1650 ms. (a) Hadamard-8 matrix with seven blocks of 500 ms each with an additional 500 ms delay before start of the readout module. (b) Hadamard-8 ‘free lunch’ matrix with a perfusion block of 2000 ms and six blocks of 250 ms each with an additional 500 ms delay before start of readout. (c) Hadamard-8 ‘T1-compensated’ matrix with seven blocks of respectively 1175, 683, 482, 374, 305, 257 and 224 ms with an additional 500 ms delay before start of readout. Block duration selected based upon T1-compensation with a T1 of 1650 ms. (d) Result of summing sub-boli 1–4 from the equidistant (traditional) Hadamard-8 matrix (a). (e) The first sub-bolus image of the free lunch approach (b) for comparison purposes.

Since the SNR of the reconstructed sub-bolus image is equal to a traditional ASL-scan with the same duration and the same PLD as the sub-bolus block, a short sub-bolus played out early in the labelling module will provide insufficient SNR to obtain a decent ASL-image. An example of this pitfall is provided in Figure 4(a) in which a Hadamard-8 matrix is acquired with all sub-boli having an equal duration of 500 ms in combination with a 500 ms PLD before start of the readout module. Whereas the sub-boli labelled shortly before imaging provide decent image quality, the SNR of the first labelled sub-bolus is insufficient. This is not a big surprise, since the SNR of this image will be equal to a traditional ASL-scan with 500 ms labelling duration and a 3500 ms PLD. It is clear that such settings do not result in an adequate SNR of the ASL-image.

Since the first labelled spins will suffer more from T1-relaxation than the last-labelled spins, the reconstructed ASL-image of the first block would have a much smaller SNR than the image of the last block, when the duration of both blocks has been chosen equal. Therefore, a frequently applied approach is to set the durations of the first block longer than that of the subsequent blocks. A typical approach is to configure the duration and PLD of the first sub-bolus similar to that of a standard pCASL-scan, e.g. a duration and PLD of 2000 ms; subsequently, the long PLD can be used for the remaining sub-boli, as shown in Figure 4(b) (the ‘Free lunch approach’, since the SNR of the reconstructed perfusion image of the first block is equal to the traditional ASL-scan with the same total scan time). A more sophisticated approach is to choose the block durations such that the amount of signal of each block at the time of imaging is equal. Such a T_1_-compensated labelling scheme is shown in Figure 4(c).^64^ In the reconstructed sub-boli images, it can be appreciated that the signal is rather constant except for the first two sub-boli. This can be explained by the fact that for these two sub-boli the label has already entered the extravascular space and will have therefore decayed for some time with the shorter T_1_ of tissue as compared to the blood T_1_, whereas the block durations were set based on a single T_1_ equal to the T_1_ of blood.

### Pitfall 2: Trying to correct for sub-optimal sub-boli durations and PLD in post-processing

Referring back to the data from Figure 4(a), one might naively try to compensate for the low SNR of the first sub-bolus, by e.g. adding up the first four sub-boli images: one could argue that this would resemble a traditional ASL-scan with 2000 ms labelling duration and a PLD of 2000 ms, which should provide sufficient SNR. However, this does, unfortunately, not work.^64^ When working out how this summing of sub-boli would be when formulated in the originally acquired data, it becomes clear that not all acquired data are included when summing the first four sub-boli and that the images that are included are not all weighted in the same manner (see Figure 4(d)). This implies that the noise in the originally acquired data will not be averaged efficiently, and especially the noise in acquisition A will be dominant in the summed image, since it is weighted by a factor 4 (note that acquisitions B, C and D are not used when the first four sub-boli images are summed). When the goal is to obtain a perfusion map with labelling duration of 2000 ms and a PLD of 2000 ms, this should be implemented by setting the duration of the first block to 2000 ms and subsequently the PLD can be exploited for the monitoring the inflow of the labelled spins by e.g. acquiring six blocks of 250 ms and then a pause of 500 ms before the start of imaging. This approach with one perfusion block and sampling of the inflow of label, as shown in Figure 4(b), was dubbed ‘free lunch’ approach by Teeuwisse et al.^64^

### Pitfall 3: Different influence of motion and other sources of artefacts on the ASL-images

Compared to traditional ASL, te-pCASL has a longer temporal footprint: whereas traditional ASL consists of two acquisitions (label and control) that are subsequently averaged, te-pCASL depends typically on 8 or 12 acquisitions (depending on the rank of the Hadamard matrix). This larger number of acquisitions combined in the reconstruction of the sub-boli ASL-images implies that when one of these acquisitions carries an artefact, it will affect all sub-boli images. Moreover, when the sub-boli images have very different contrast, such as high contrast angiographic signal at short PLDs and low contrast perfusion signal at long PLDs, then a shine-through of angiographic information in the perfusion images may occur. The different way that artefacts can affect the perfusion images should be taken into account when employing te-pCASL in a clinical setting. The encoding procedure can be made more resistant to motion artefacts by changing from a Hadamard matrix to a Walsh-type of encoding.^65^ Walsh-encoding already enables decoding temporal information, albeit at a lower temporal resolution, while the matrix has not yet been completely acquired. This would enable a premature end of the acquisition, e.g. due to restlessness of the patient, while still being able to recover some dynamic information. Also on the post-processing site improvements to increase the robustness against motion artefacts have been proposed. For example, Von Samson-Himmelstjerna et al. have proposed a Bayesian decoding scheme that allows for detection and disregarding of acquisitions that add more artefacts to the quantitative CBF- and ATT-maps than signal.^66^

### Example 1: Measuring label dynamics I: Combined CBF and ATT mapping

The most traditional application of te-pCASL is to apply the free lunch approach to quantify the CBF without an SNR-penalty, while measuring the ATT at the same time.^64,67,68^ The first sub-bolus is chosen similarly to a traditional pCASL-sequence and the PLD-time is filled with additional sub-boli to monitor the inflow of labelled spins. Based on this data, normal ASL-post-processing can be employed to simultaneously estimate CBF and ATT.

### Example 2: Quick survey of haemodynamic status

The advantage of highly efficient acquisition of information can be exploited to the fullest when employing it as a ‘haemodynamic survey’ to assess quickly the haemodynamic status of the studied subject. By using a resolution that is much lower than usual for the readout module, the SNR is increased, so that a single average is sufficient. A fit of the kinetic curve can subsequently yield an estimate of the ATT, based on which the PLD of the subsequent normal resolution ASL-scan can be based. This approach has been implemented by Dai et al.^68^ Main issue when employing such an approach in a research setting would be that the employed PLD would be different for each and every subject, which might complicate interpretation, i.e. due to the inhomogeneous acquisition settings. It can, however, also be argued that the measured information will be *more* homogeneous between the different subjects, since the readout is timed to correct for inter-individual differences in arterial transit time. Which of these two possible effects would be dominant in a clinical research setting should be further tested.

### Example 3: Measuring label dynamics II: Spin compartment measurement

While normally in ASL ‘just’ the presence of label is detected by means of a fast, proton-density weighted readout sequence, one can also sensitize the readout to measure other properties of the labelled water protons, such as the local T_2_-value. This can be done by including a global T_2_-preparation just before the readout. Wells et al. and Liu et al. were among the first to realize that this approach can be employed to measure the water transition times, i.e. the moment that the labelled spins move from the intra- to the extravascular compartment.^69,70^ This physiological parameter is first of all interesting for ASL-quantification, since it determines when the label will start to decay with the T_1_ of the extravascular compartment instead of the T_1_ of blood. However, it is also likely that this transition time will become shorter when the blood-brain-barrier (BBB) is damaged and that changes in transition time would already be detectable for rather subtle BBB-breakdown. A disadvantage of those measurements is that they are time-consuming when performed by consecutively measuring ASL-scans with different labelling durations and PLD. However, by doing this in a time-encoded fashion, such measurements can be performed in approximately 10 min, making it a feasible option for research protocols, especially when taking into account that contrast agent based methods take approximately 15–25 min.^71,72^

### Example 4: Combining two ASL-contrasts that rely on different timing of the labelling

When two (or more) different labelling durations and PLDs are needed for different scans, one could consider combining these into one single scan as long as the readout modules are not overlapping. As an example, one could combine a normal perfusion ASL-scan (e.g. 1800 ms labelling and 1800 ms PLD) with a labelling efficiency scan (labelling duration around 800 ms, PLD as short as possible). The complete labelling efficiency scan can in this example be acquired during the PLD of the traditional scan.^73^ This approach works, because the readout of the labelling efficiency scan can be performed only a little bit superior to the labelling plane and can therefore still be performed below the imaging stack of the perfusion scan. When the readout for labelling efficiency would overlap with the imaging slices of the perfusion scan, it would affect the signal from the perfusion measurement, which would (locally) lower the detected perfusion signal and could therefore create regional variation in ASL-signal intensities.

### Example 5: Change the encoded information for a single sub-bolus

Due to the low intrinsic SNR of ASL even for time-encoded pCASL multiple averages are needed. For these different repeats, one could change the labelling modus for a single sub-bolus to encode even more information into the sequence, such as a vessel-selective labelling modus. When doing this for the second sub-bolus (i.e. employ the first sub-bolus for perfusion imaging), one can add a flow territory map to the CBF- and ATT-map as traditionally obtained from a te-pCASL-dataset.^74^ Risk in this approach is that the labelled spins have not reached the border-zones of the flow territories during the second sub-bolus, which would seriously limit the value of this approach (the global flow territories can be well predicted from the Circle-of-Willis configuration, while the main individual changes are in the exact location of the border-zones).^75,76^

### Example 6: Combine te-pCASL with a dual readout module

**Figure 5.**
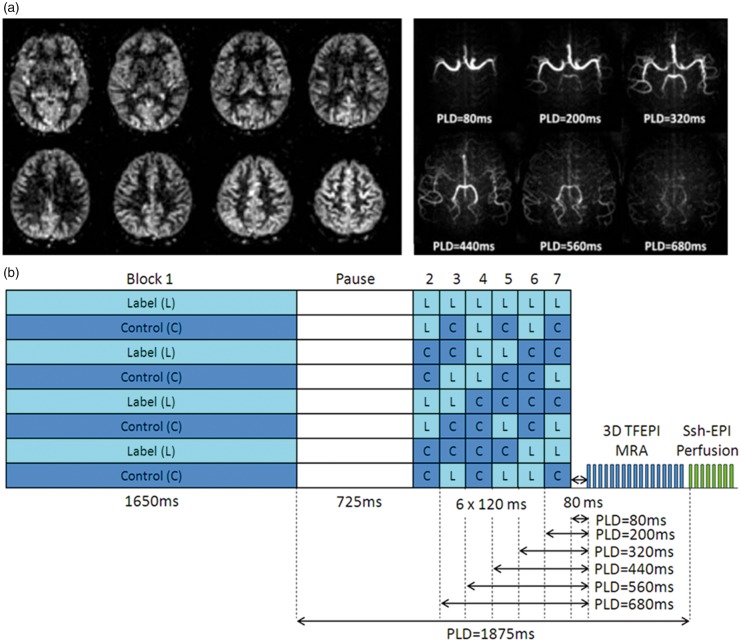
Combined 4D-ASL-angiography and perfusion imaging. (a) A subset of slices are depicted of the perfusion part of the sequence (acquired with a PLD of 1875 ms) as well as maximum intensity projection images of the angiography readout at multiple PLDs. (b) The encoding scheme in which a ‘free-lunch’-like Hadamard encoding matrix is combined with a dual readout approach. The segmented 3D TFEPI readout module consumes approximately 20% of the available ASL-signal, leading to a 20% lower SNR of the perfusion scan as compared to a perfusion only scan. This small drop in SNR was counteracted by slightly decreasing the spatial resolution.

A basic characteristic of any pCASL-scan is that most time is invested in the labelling and PLD-modules: typically 3.5–4 s of a pCASL-scan is used for labelling and PLD, while only 300–900 ms is employed for readout of the labelled signal. This stresses the importance of extracting as much information as possible from each pCASL-labelling module. One way of extracting more information is to dedicate a small percentage of the labelled magnetization to an additional readout module. As an example, the low resolution readout module as employed for perfusion ASL can be preceded by a high spatial resolution readout module for ASL-angiography.^77^ By filling the PLD of the perfusion scan with additional sub-boli at shorter PLD-timings, 4D-MRA images can be recorded at a high spatial resolution (see Figure 5).

In conclusion, time-encoded pCASL offers a huge amount of flexibility into a normal ASL-scan and many future applications can be envisioned.

## Advances in readout strategy

MRI has seen highly significant innovations in readout strategies in recent years. Four of these developments are particularly interesting for ASL-imaging and will be discussed in more detail below.

### Golden angle radial acquisition

**Figure 6.**
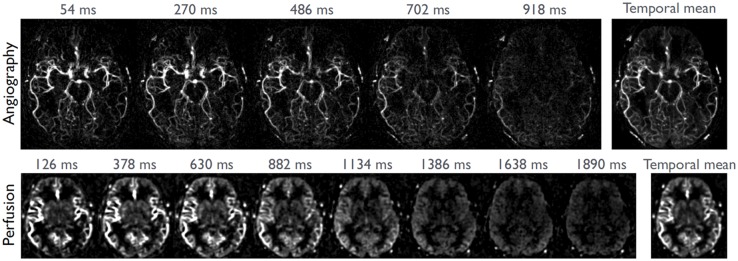
Combined angiography and perfusion using radial imaging and ASL (CAPRIA) as based upon the Golden Angle Radial approach. Selected angiographic frames are shown from a transverse maximum intensity projection (108 ms temporal resolution, top) as well as a single slice of perfusion data (252 ms temporal resolution, bottom), reconstructed from the same raw data sets. Image courtesy: Thomas Okell (FMRIB Centre, Nuffield Department of Clinical Neurosciences, University of Oxford, Oxford, UK).

The golden angle radial acquisition approach offers several interesting features for ASL.^78^ First of all, the continuous acquisition provides highly flexible dynamic reconstruction opportunities, enabling sliding window reconstructions to achieve a better temporal sampling of the dynamic ASL-signal. Second, the trade-off between temporal and spatial resolution can easily be exploited during post-processing enabling for example high spatial resolution ASL-MRA images as well as lower resolution perfusion images. Importantly, combination of spokes can both be performed with spokes acquired directly after the same labelling module (i.e. these spokes will have slightly different PLD), or with spokes acquired after different repeats of the labelling module (i.e. spokes with the same PLD), whereas most of the times a combination of these two approaches will be chosen. Third, one can imagine that this approach will provide excellent possibilities of motion artefact suppression, especially since each readout covers the centre of k-space. Motion artefact suppression could either be done by excluding motion affected spokes or by continuous estimation of motion and real-time adoption of acquisition geometries, similar to prospective motion correction.^79,80^ Figure 6 shows an example of the versatility of employing a golden angle radial readout within an ASL-sequence, thereby enabling the reconstruction of both angiography and perfusion images from the same raw data-sets when acquired with the ‘Combined Angiography and Perfusion using Radial Imaging and ASL (CAPRIA)’-approach.^81^

### Simultaneous multislice (SMS) or multiband

In recent years, the technique of SMS, also known as multiband, has dramatically improved the acquisition performance of BOLD and DTI-sequence as especially exploited within the neuroscience community. With the SMS-technique, multiple slices can be acquired simultaneously without significant SNR-penalties (see Barth et al.^82^ for a review of this technique). For ASL, SMS has three important advantages all stemming from the shorter readout time.^83–86^ First of all, it enables a much larger coverage of the brain for especially Look-Locker-based multi-PLD ASL-sequences. The limitation of traditional readout approaches is evident from e.g. the frequently employed QUASAR-technique, which typically only measures seven slices of 6 mm thickness.^61^ Whole brain coverage could easily be achieved, just by including a multiband-factor of three into the same readout-module. Second, SMS can provide much more constant and thus on average better background suppression as well as more constant PLD for different slices in multi-slice ASL than traditional readout techniques. For a traditional multi-slice, single-shot EPI-readout approximately 35 ms is required per slice when in-plane parallel imaging is employed.^51^ This implies that when e.g. 20 slices are acquired, the PLD of the last slice will be 665 ms longer than the PLD of the first slice. Moreover, in those 665 ms, approximately 42% of the gray matter magnetization will have recovered, resulting in deteriorated background in the last acquired slices. Both effects will add together and degrade the SNR significantly in these slices. Finally, due to the more efficient sampling within the readout module, the TR of the ASL-sequence can become shorter allowing more averages within the same scan-time. One should keep in mind, however, that the ASL-preparation (labelling and PLD) take-up much more time than the readout-part of the sequence, making the TR-advantages of SMS relatively minor for ASL.

**Figure 7.**
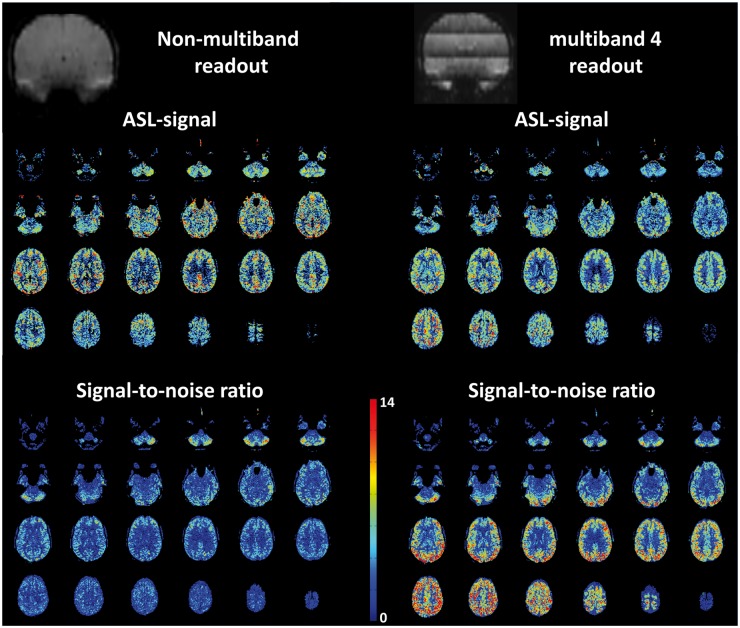
Example of the potential of multiband (also known as simultaneous multi-slice (SMS) acquisition) for ASL (scans acquired on a 3 T MRI scanner (Ingenia, Philips, The Netherlands; scanner software was locally adapted to allow multiband ASL) on a 43-year-old, male volunteer who provided informed consent under an approved IRB-protocol for protocol development). Left: traditional readout with single-shot EPI (half Fourier of 0.8; readout time per slice 60 ms; 24 slices; 1800 ms labelling duration; 1800 ms PLD; two background suppression inversion pulses; 30 averages; total scan time 5m08s). Right: same settings except for multiband factor of 4; 38 averages; total scan time 5m11s. Upper row: coronal reconstruction of mean control scans showing a gradual decrease in background suppression efficiency for the traditional readout (Left) and four bands of decreasing background suppression efficiency for the multiband-4 readout (Right). Middle row: ASL-images. Lower row: SNR images calculated as ASL-signal divided by standard deviation over the acquired averages multiplied by the square root of the number of averages to achieve the effective SNR corrected for the shorter achievable TR for multiband readout as compared to the traditional readout.

An extreme example of the potential advantages of multiband ASL is illustrated in Figure 7, where a readout of 24 slices without SMS or in-plane parallel imaging (readout time 60 ms per slice) is compared with the same readout, but with a multiband factor of 4. This example clearly demonstrated the more homogenous SNR-distribution for the multiband readout, especially for the last acquired slices.

### Fingerprinting ASL

The revolutionizing new approach of MR-Fingerprinting that pseudo-randomly varies sequence parameters in combination with fast, single shot spiral readouts,^87^ has also been applied to ASL in a few feasibility studies.^88,89^ These studies all employ randomization of the labelling duration and PLD, sometimes in combination with random ordering of label and control conditions, i.e. not strictly interleaving. A final important feature of the proposed implementations is that readout flip angles smaller than 90° are employed and that no saturation of brain tissue is performed before the start of the labelling module. This implies that a built-up of label can occur in the brain tissue, thereby increasing the perfusion information content. By randomizing the labelling duration and the PLDs in combination with a dictionary obtained from well-known ASL-models,^49^ several parameters can be estimated from a single scan, such as CBF, ATT, B_1_ and T_1_. The current approach inherited several features of the Pseudo-Random Arterial Modulation (PRAM) MRI-technique, which also employed a randomization of label and control modules, but employed a deconvolution type of reconstruction instead of the dictionary approach.^90^ It will be interesting to see how much more information can be inferred from such Fingerprinting ASL-experiments when more variation in the readout module is included, such as different echo times, crusher strengths, etc.

### Compressed sensing

Compressed sensing^91^ has gained relatively little interest for use in ASL-sequences, probably mainly explained by the low SNR of ASL as well as the fact that most common readout strategies, such as 3D-GRASE, EPI and 3D stack-of-spirals, employ a single excitation pulse and fast readouts covering a large part of k-space. One study employed compressed sensing to increase the spatial coverage of balanced steady-state free precession (bSSFP) readouts.^92^ Whereas another study employed compressed sensing on multi-timepoint ASL-data with a reconstruction based upon a model of potential perfusion time courses.^93^ However, with the introduction of Golden Angle Radial and Fingerprinting approaches into ASL-sequences, it can be expected that also compressed sensing approaches will be employed more frequently as a crucial step in efficient reconstruction of sparse data.

## Motion sensitizing gradients for perfusion information

Already in the 80s Le Bihan recognized the ability of diffusion gradients to provide information on microvascular blood flow.^13^ The technique proposed by Le Bihan, IVIM, measures diffusion-weighted MRI-signal over a wide range of b-values to discriminate the microvascular component with a high apparent diffusion coefficient (ADC) from true diffusion, which has a much smaller ADC. This can be done by e.g. fitting a bi-exponential function through the MR-signal as a function of b-value. Whereas applications in the brain have been relatively scarce in the following 30 years, recently an increased interest in IVIM can be observed, probably mainly driven by improved and faster diffusion performance of modern MRI-scanners. Moreover, the idea of employing motion-sensitizing gradients is also at the heart of velocity and acceleration selective ASL (respectively VS-ASL and AccASL).^94,95^ Finally, motion sensitizing gradients can be employed within the ASL-sequence to obtain more detailed information on the structure of the microvasculature.^96,97^

### Non-spatially selective ASL

Wong proposed the use of a velocity-selective preparation module as a way to generate label not only in the large brain feeding arteries below the imaging volume as done in traditional spatially selective ASL, but also *within* the imaging volume of an ASL-experiment.^94,98–100^ The main advantage of this approach is that it dramatically reduces the transit time from the place where the ASL-label is created to the moment that the label arrives in the microvasculature. As a control condition the same module, but without the motion sensitizing gradients is used. This implies that VS-ASL, similar to other ASL-methods, relies on a subtraction to cancel out static brain tissue signal, which can also be considered the main difference with the earlier mentioned IVIM-approach. When employing a velocity selective module, two main issues arise. First of all, not only arterial blood will be labelled, but also venous blood. Second, it is undetermined how much label is created, which makes quantification of CBF challenging. Wong came up with an approach that solves both issues at the same time, by proposing the inclusion of a second velocity sensitizing module just before imaging with the same cut-off velocity as the first one. The time between the two velocity encoding modules is chosen similar to the PLD of a normal ASL-experiment. Venous blood will be suppressed by the second module, because venous blood will increase its velocity when collecting in the larger veins. Moreover, this approach fixates the temporal width of the label bolus, similar to the QUIPSS-II technique,^101^ to the time between the two velocity selective modules.

**Figure 8.**
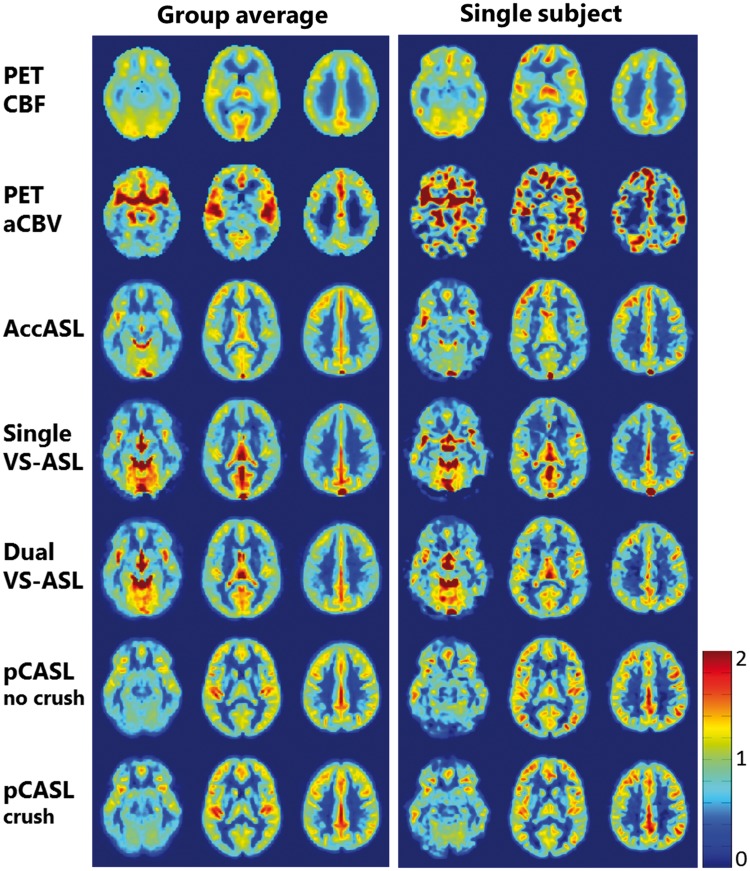
Comparison of acceleration-selective and velocity-selective ASL-maps with [^15^O]H_2_O PET for three transversal slices. Both the group average (N = 13) and a single subject data-set are shown. For comparison of the spatial distribution of the signal, all maps were normalized by dividing each voxel by the average gray matter value of the corresponding map. AccASL: acceleration-selective arterial spin labelling; aCBV: arterial cerebral blood volume; CBF: cerebral blood flow; pCASL: pseudo-continuous ASL; PET: positron emission tomography; VS-ASL: velocity-selective ASL. Image reproduced with permission from Schmid et al.^95^

As an alternative for distinguishing arterial from venous label, acceleration selective ASL has been proposed.^95^ In this approach, labelling is based upon acceleration or deceleration of blood. Changes in blood velocity occur both when the blood traverses through the vascular tree, during the cardiac cycle as well as due to tortuosity of the vessels (i.e. due to a change in the directionality of blood flow). Since most of these effects are predominantly occurring on the arterial side of the microvasculature, this approach mainly labels arterial blood. Figure 8 shows a comparison between VS-ASL, AccASL, pCASL and [^15^O]H_2_O PET in young volunteers.

Interestingly, motion sensitizing gradients can also be employed to infer information from the structure of the microvasculature. This can be performed, similar to diffusion tensor imaging, by changing the direction of the motion-sensitizing gradients in the velocity selective labelling module,^102^ but maybe even more interesting is the idea to combine traditional ASL-approaches with flow crushers in different orientations.^96^ By employing the first approach, Frank et al. were able to prove a higher fractional anisotropy for CBF at higher velocities, which is in agreement with our understanding of the cerebral vasculature. With the second approach, it is possible to obtain information on the direction of the blood flow at a certain PLD. In this approach, the PLD is seen as a sequence parameter that allows to aim for a certain location within the vascular tree. By prolonging the PLD, the change in directionality of the vasculature can be tracked, which can be an important source of insight into the microvascular architecture and potentially enable to measure microvascular tortuosity.

Finally, Luo and Hernandez Garcia proposed a small modification of the velocity selective method that can encode the distribution of blood flow velocity into the signal of a VS-ASL experiment.^103^ This could potentially pave the way to measure newly proposed physiological parameters such as capillary transit times heterogeneity (CTTH).^104,105^

It can be expected that all these techniques will see important development in the next few years and that they will finally enable to assess in vivo the microvascular architecture, such as tortuosity, and thereby allows us to study how the microvascular architecture is influenced by (or is a causative factor of) neurodegenerative diseases.

## Flow territory mapping

Since ASL is based on local inversion of inflowing blood and the subsequent detection of the labelled spins downstream within the cerebral vascular tree and/or its accumulation into the tissue compartment, it also enables the depiction of flow territories, or sub-parts of the vascular tree, when labelling is restricted to one (or several) brain-feeding arteries. The only other medical modality that can selectively image a single artery is dynamic subtraction angiography (DSA), which is based on iodinated contrast agent injection in the targeted artery by means of an inserted catheter. DSA is therefore much more invasive than selective ASL, which makes the latter a perfect candidate for both more fundamental research on remodelling of the cerebral vascular tree in cerebrovascular diseases or as part of the work-up towards an interventional procedure. As part of the work-up, the interventional radiologist can already use vessel-selective ASL to gain insight into the vascular structure that will be encountered and optimize the intervention and treatment strategy.

Three main techniques for vessel-selective ASL have been proposed. The first is based on angulation of the thick labelling slab of a pulsed ASL (PASL) sequence; the second is based upon several acquisitions with different spatial labelling efficiency distributions within the labelling plane in combination with post-processing to identify flow territories (vessel-encoded pCASL or ve-pCASL); finally, the third approach is based upon a rotating in-plane gradient during a pCASL-labelling train that results in a circular (or ellipsoid) labelling spot that enables the (super)selective labelling of a single artery. The first approach was the first method to enable flow territory mapping and important, proof-of-principle studies have been performed utilizing this approach. These studies have confirmed that the configuration of the Circle-of-Willis has profound influence on the flow territory distribution of the internal carotid arteries and the posterior circulation.^106,107^ However, considerable differences remain when looking into subjects with the same configuration of the Circle-of-Willis. Furthermore, it demonstrated that the architecture of the vascular tree undergoes remodelling in large vessel disease patients.^75^ Still, this approach has some important limitations. First of all, the planning process is rather cumbersome, since the thick labelling slab should exclusively hit the targeted vessel without creating contaminating signal in other brain feeding arteries. Second, the baseline SNR of PASL-sequences is lower than those of pCASL. Therefore, it can be expected that the other two sequences, both based on pCASL, will become the more commonly employed sequences for future applications. Both have their own, distinct advantages. ve-pCASL enables a virtually planning-free approach, since the only requirement is that the applied variation in spatial distribution of labelling efficiency sufficiently differs for the arteries that one wants to differentiate.^108,109^ An adequately elaborate labelling scheme can therefore have enough flexibility to discern the sought-after flow territories. Main hurdle for direct application in a clinical setting is the necessity of advanced post-processing procedures, which are currently only available off-line. Furthermore, in complex vasculature, this approach might create some uncertainty whether sufficient discriminating power was present in the acquisition and thus whether the obtained flow territory maps are trustworthy. This can partly be solved by approaches that allow to back-project the estimated source location of the found flow territory onto the labeling plane.^110^ When these source locations do not coincide with the location of arteries, one can be sure that the observed flow territories are inaccurate. Such a ‘feedback’-mechanism can therefore provide sufficiently solid proof to the clinician that the targeted flow territory has indeed been identified correctly. The super-selective approach provides in a relatively shorter time-frame the supplied tissue of a single artery.^111,112^ Since the labelling exclusively labels a single artery, less encoding time is needed as for ve-pCASL in which multiple vessels have to be decoded. Furthermore, the close resemblance between the super-selective approach with the DSA-technique and the fact that post-processing can easily be done on the MR-console (similar to normal pCASL), makes this an attractive approach for early adoption by clinicians. On the downside, the super-selective approach relies on careful planning of the labelling spot, which for complicated vasculature such as arterio-venous malformations (AVMs) may require the clinician to be present during planning of the data.

**Figure 9.**
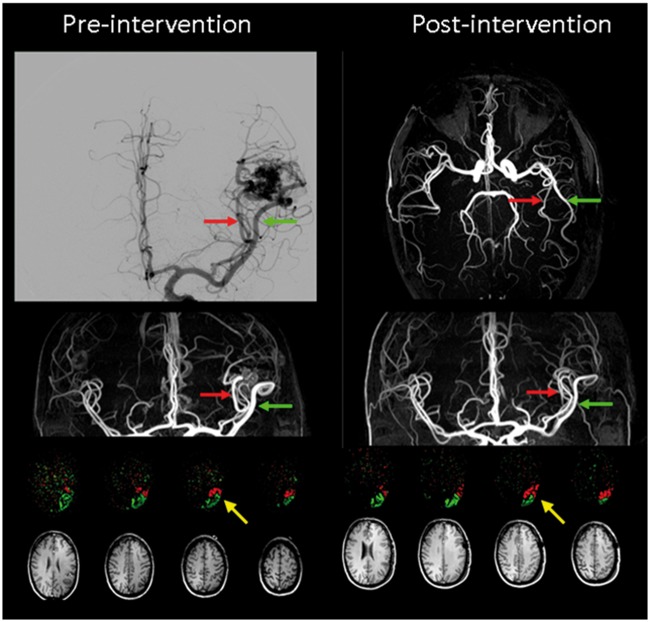
Super-selective flow territories of two feeders (indicated with red and green arrows; perfusion territories are indicated in the same colours) of an arterio-venous malformation (AVM) in the left temporal lobe in a 48-year-old male patient; Left before and right after surgical removal of the AVM. Corresponding T_1_-weighted images are shown as anatomical reference. After surgery significant changes in the perfusion territories of the feeders can be observed, see e.g. the yellow arrow. Images reproduced with permission from Helle et al.^113^

Figure 9 illustrates the potential of flow territory mapping for planning and evaluation of cerebral interventions (adopted with permission from Helle et al.^113^). Pre-intervention imaging shows that the two large feeding vessels of the AVM feed a considerable part of the cortex, clearly demonstrating that occlusion of the AVM has to be performed further downstream. Post-intervention flow territory mapping shows that part of the cortex is now fed by the other feeding artery, showing that by surgical operation of the AVM a redistribution of blood flow occurred. Interestingly, the part of the cortex that changed from one feeder to the other was proven by fMRI to be Wernicke’s area, which may explain the development of speech difficulties in this patient. These speech problems subdued within a few weeks, which might be caused by a gradual adaptation to the remodelling of the vascular supply.

## Concluding remarks

Although with the publication of the consensus paper in 2015, an important step has been taken towards clinical acceptance of ASL, remaining issues as well as newly identified opportunities have led to a considerable set of technological innovations of ASL. Whereas some, like flow territory mapping and velocity-selective ASL, have already been applied for a longer period of time in research studies and for which the important next step would be general availability on clinical scanners, others like Fingerprinting ASL were just recently proposed and have only been tested in pilot studies. As a general trend in the newly taken avenues in ASL, one could point to the general aim of obtaining more information than just CBF from ASL-scans without considerable increases in scan-time. This holds true for time-encoded ASL, which allows for highly efficient dynamic ASL-measurements, as well as for other approaches, such as Golden Angle radial that allow combined 4D-angiography and perfusion imaging. With these new developments showing great promise, it is important that the consensus statement is seen as a reflection of the state-of-the-art in 2014 and that further updates should be amended in the near future.

## References

[1] Raichle ME Neuroscience. The brain's dark energy. Science 2006; 314: 1249–1250. 17124311

[2] Saver JL Time is brain–quantified. Stroke 2006; 37: 263–266. 16339467 10.1161/01.STR.0000196957.55928.ab

[3] Astrup J Siesjo BK Symon L Thresholds in cerebral ischemia - the ischemic penumbra. Stroke 1981; 12: 723–725. 6272455 10.1161/01.str.12.6.723

[4] Fatterpekar GM Galheigo D Narayana A et al. Treatment-related change versus tumor recurrence in high-grade gliomas: a diagnostic conundrum–use of dynamic susceptibility contrast-enhanced (DSC) perfusion MRI. AJR Am J Roentgenol 2012; 198: 19–26. 22194475 10.2214/AJR.11.7417

[5] Law M Yang S Wang H et al. Glioma grading: sensitivity, specificity, and predictive values of perfusion MR imaging and proton MR spectroscopic imaging compared with conventional MR imaging. AJNR Am J Neuroradiol 2003; 24: 1989–1998. 14625221 PMC8148904

[6] Shiroishi MS Castellazzi G Boxerman JL et al. Principles of T2*-weighted dynamic susceptibility contrast MRI technique in brain tumor imaging. J Magn Reson Imaging 2015; 41: 296–313. 24817252 10.1002/jmri.24648

[7] Phillips AA Chan FH Zheng MM et al. Neurovascular coupling in humans: Physiology, methodological advances and clinical implications. J Cereb Blood Flow Metab 2016; 36: 647–664. 26661243 10.1177/0271678X15617954 PMC4821024

[8] Ogawa S Lee TM Kay AR et al. Brain magnetic resonance imaging with contrast dependent on blood oxygenation. Proc Natl Acad Sci USA 1990; 87: 9868–9872. 2124706 10.1073/pnas.87.24.9868 PMC55275

[9] Kwong KK Belliveau JW Chesler DA et al. Dynamic magnetic resonance imaging of human brain activity during primary sensory stimulation. Proc Natl Acad Sci USA 1992; 89: 5675–5679. 1608978 10.1073/pnas.89.12.5675 PMC49355

[10] Alsop DC Detre JA Reduced transit-time sensitivity in noninvasive magnetic resonance imaging of human cerebral blood flow. J Cereb Blood Flow Metab 1996; 16: 1236–1249. 8898697 10.1097/00004647-199611000-00019

[11] Detre JA Leigh JS Williams DS et al. Perfusion imaging. Magn Reson Med 1992; 23: 37–45. 1734182 10.1002/mrm.1910230106

[12] Williams DS Detre JA Leigh JS et al. Magnetic resonance imaging of perfusion using spin inversion of arterial water. Proc Natl Acad Sci USA 1992; 89: 212–216. 1729691 10.1073/pnas.89.1.212 PMC48206

[13] Le Bihan D Breton E Lallemand D et al. MR Imaging of intravoxel incoherent motions: application to diffusion and perfusion in neurologic disorders. Radiology 1986; 161: 401–407. 3763909 10.1148/radiology.161.2.3763909

[14] Lu H Golay X Pekar JJ et al. Functional magnetic resonance imaging based on changes in vascular space occupancy. Magn Reson Med 2003; 50: 263–274. 12876702 10.1002/mrm.10519

[15] Gorelick PB Scuteri A Black SE et al. Vascular contributions to cognitive impairment and dementia: a statement for healthcare professionals from the american heart association/american stroke association. Stroke 2011; 42: 2672–2713. 21778438 10.1161/STR.0b013e3182299496 PMC3778669

[16] Stanimirovic DB Friedman A Pathophysiology of the neurovascular unit: disease cause or consequence? J Cereb Blood Flow Metab 2012; 32: 1207–1221. 22395208 10.1038/jcbfm.2012.25 PMC3390807

[17] Iturria-Medina Y Sotero RC Toussaint PJ et al. Early role of vascular dysregulation on late-onset Alzheimer's disease based on multifactorial data-driven analysis. Nat Commun 2016; 7: 11934. 27327500 10.1038/ncomms11934 PMC4919512

[18] Xekardaki A Rodriguez C Montandon ML et al. Arterial spin labeling may contribute to the prediction of cognitive deterioration in healthy elderly individuals. Radiology 2015; 274: 490–499. 25291458 10.1148/radiol.14140680

[19] Grubb RL Jr. Derdeyn CP Fritsch SM et al. Importance of hemodynamic factors in the prognosis of symptomatic carotid occlusion. JAMA 1998; 280: 1055–1060. 9757852 10.1001/jama.280.12.1055

[20] van Laar PJ Hendrikse J Klijn CJ et al. Symptomatic carotid artery occlusion: flow territories of major brain-feeding arteries. Radiology 2007; 242: 526–534. 17255422 10.1148/radiol.2422060179

[21] van Laar PJ Hendrikse J Mali WP et al. Altered flow territories after carotid stenting and carotid endarterectomy. J Vasc Surg 2007; 45: 1155–1161. 17543680 10.1016/j.jvs.2006.11.067

[22] Ostergaard L Sorensen AG Kwong KK et al. High resolution measurement of cerebral blood flow using intravascular tracer bolus passages. Part II: Experimental comparison and preliminary results. Magn Reson Med 1996; 36: 726–736. 8916023 10.1002/mrm.1910360511

[23] Ostergaard L Weisskoff RM Chesler DA et al. High resolution measurement of cerebral blood flow using intravascular tracer bolus passages. Part I: Mathematical approach and statistical analysis. Magn Reson Med 1996; 36: 715–725. 8916022 10.1002/mrm.1910360510

[24] Rempp KA Brix G Wenz F et al. Quantification of regional cerebral bloodflow and volume with dynamic susceptibility contrast-enhanced MR Imaging. Radiology 1994; 193: 637–641. 7972800 10.1148/radiology.193.3.7972800

[25] Belliveau JW Rosen BR Kantor HL et al. Functional cerebral imaging by susceptibility-contrast NMR. Magn Reson Med 1990; 14: 538–546. 2355835 10.1002/mrm.1910140311

[26] Detre JA Leigh JS Williams DS et al. Perfusion imaging. Magn Reson Med 1992; 23: 37–45. 1734182 10.1002/mrm.1910230106

[27] Alsop DC Detre JA Golay X et al. Recommended implementation of arterial spin-labeled perfusion MRI for clinical applications: A consensus of the ISMRM perfusion study group and the European consortium for ASL in dementia. Magn Reson Med 2015; 73: 102–116. 24715426 10.1002/mrm.25197 PMC4190138

[28] Heijtel DF Mutsaerts HJ Bakker E et al. Accuracy and precision of pseudo-continuous arterial spin labeling perfusion during baseline and hypercapnia: A head-to-head comparison with O^15^H_2_O positron emission tomography. Neuroimage 2014; 92C: 182–192. 10.1016/j.neuroimage.2014.02.011 24531046

[29] Zhang K Herzog H Mauler J et al. Comparison of cerebral blood flow acquired by simultaneous [15O]water positron emission tomography and arterial spin labeling magnetic resonance imaging. J Cereb Blood Flow Metab 2014; 34: 1373–1380. 24849665 10.1038/jcbfm.2014.92 PMC4126098

[30] Kilroy E Apostolova L Liu C et al. Reliability of two-dimensional and three-dimensional pseudo-continuous arterial spin labeling perfusion MRI in elderly populations: comparison with 15O-water positron emission tomography. J Magn Reson Imaging 2014; 39: 931–939. 24038544 10.1002/jmri.24246 PMC3866214

[31] Musiek ES Chen Y Korczykowski M et al. Direct comparison of fluorodeoxyglucose positron emission tomography and arterial spin labeling magnetic resonance imaging in Alzheimer’s disease. Alzheimers Dement 2012; 8: 51–59. 22018493 10.1016/j.jalz.2011.06.003 PMC3264701

[32] Dai W Garcia D de Bazelaire C et al. Continuous flow-driven inversion for arterial spin labeling using pulsed radio frequency and gradient fields. Magn Reson Med 2008; 60: 1488–1497. 19025913 10.1002/mrm.21790 PMC2750002

[33] Jung Y Wong EC Liu TT Multiphase pseudocontinuous arterial spin labeling (MP-PCASL) for robust quantification of cerebral blood flow. Magn Reson Med 2010; 64: 799–810. 20578056 10.1002/mrm.22465

[34] Luh WM Talagala SL Li TQ et al. Pseudo-continuous arterial spin labeling at 7 T for human brain: estimation and correction for off-resonance effects using a Prescan. Magn Reson Med 2013; 69: 402–410. 22488568 10.1002/mrm.24266 PMC3402610

[35] Jahanian H Noll DC Hernandez-Garcia L B(0) field inhomogeneity considerations in pseudo-continuous arterial spin labeling (pCASL): effects on tagging efficiency and correction strategy. NMR Biomed 2011; 24: 1202–1209. 21387447 10.1002/nbm.1675

[36] Zhao L, Vidorreta M, Soman S, et al. Improving the robustness of pseudo-continuous arterial spin labeling to off-resonance and pulsatile flow velocity. *Magn Reson Med*. Epub ahead of print October 23 2016. DOI: 10.1002/mrm.26513. 10.1002/mrm.26513 PMC5848499 27774656

[37] Chen Z, Zhang X, Yuan C, et al. Measuring the labeling efficiency of pseudocontinuous arterial spin labeling. *Magn Reson Med* 2017; 77: 1841–1852. 10.1002/mrm.26266 27174204

[38] Aslan S Xu F Wang PL et al. Estimation of labeling efficiency in pseudocontinuous arterial spin labeling. Magn Reson Med 2010; 63: 765–771. 20187183 10.1002/mrm.22245 PMC2922009

[39] Parkes LM Tofts PS Improved accuracy of human cerebral blood perfusion measurements using arterial spin labeling: accounting for capillary water permeability. Magn Reson Med 2002; 48: 27–41. 12111929 10.1002/mrm.10180

[40] Hales PW Kirkham FJ Clark CA A general model to calculate the spin-lattice (T1) relaxation time of blood, accounting for haematocrit, oxygen saturation and magnetic field strength. J Cereb Blood Flow Metab 2016; 36: 370–374. 26661147 10.1177/0271678X15605856 PMC4759664

[41] Lu H Clingman C Golay X et al. Determining the longitudinal relaxation time (T1) of blood at 3.0 Tesla. Magn Reson Med 2004; 52: 679–682. 15334591 10.1002/mrm.20178

[42] Li W Grgac K Huang A et al. Quantitative theory for the longitudinal relaxation time of blood water. Magn Reson Med 2016; 76: 270–281. 26285144 10.1002/mrm.25875 PMC4758918

[43] Vaclavu L van der Land V Heijtel DF et al. In Vivo T1 of blood measurements in children with sickle cell disease improve cerebral blood flow quantification from arterial spin-labeling MRI. AJNR Am J Neuroradiol 2016; 37: 1727–1732. 27231223 10.3174/ajnr.A4793 PMC7984682

[44] Gevers S Nederveen AJ Fijnvandraat K et al. Arterial spin labeling measurement of cerebral perfusion in children with sickle cell disease. J Magn Reson Imaging 2012; 35: 779–787. 22095695 10.1002/jmri.23505

[45] Oguz KK Golay X Pizzini FB et al. Sickle cell disease: continuous arterial spin-labeling perfusion MR imaging in children. Radiology 2003; 227: 567–574. 12663827 10.1148/radiol.2272020903

[46] Varela M Hajnal JV Petersen ET et al. A method for rapid in vivo measurement of blood T1. NMR Biomed 2011; 24: 80–88. 20669148 10.1002/nbm.1559

[47] Zhang X Petersen ET Ghariq E et al. In vivo blood T(1) measurements at 1.5 T, 3 T, and 7 T. Magn Reson Med 2013; 70: 1082–1086. 23172845 10.1002/mrm.24550

[48] Li W, Liu P, Lu H, et al. Fast measurement of blood T1 in the human carotid artery at 3T: Accuracy, precision, and reproducibility. *Magn Reson Med* 2017; 77: 2296–2302. 10.1002/mrm.26325 PMC5250597 27436420

[49] Buxton RB Frank LR Wong EC et al. A general kinetic model for quantitative perfusion imaging with arterial spin labeling. Magn Reson Med 1998; 40: 383–396. 9727941 10.1002/mrm.1910400308

[50] van Gelderen P de Zwart JA Duyn JH Pittfalls of MRI measurement of white matter perfusion based on arterial spin labeling. Magn Reson Med 2008; 59: 788–795. 18383289 10.1002/mrm.21515 PMC12629510

[51] van Osch MJ Teeuwisse WM van Walderveen MA et al. Can arterial spin labeling detect white matter perfusion signal? Magn Reson Med 2009; 62: 165–173. 19365865 10.1002/mrm.22002

[52] Skurdal MJ Bjornerud A van Osch MJ et al. Voxel-wise perfusion assessment in cerebral white matter with PCASL at 3T; is it possible and how long does it take? PLoS One 2015; 10: e0135596. 26267661 10.1371/journal.pone.0135596 PMC4534420

[53] Gunther M Bock M Schad LR Arterial spin labeling in combination with a look-locker sampling strategy: inflow turbo-sampling EPI-FAIR (ITS-FAIR). Magn Reson Med 2001; 46: 974–984. 11675650 10.1002/mrm.1284

[54] Hendrikse J van Osch MJ Rutgers DR et al. Internal carotid artery occlusion assessed at pulsed arterial spin-labeling perfusion MR imaging at multiple delay times. Radiology 2004; 233: 899–904. 15486211 10.1148/radiol.2333031276

[55] Kramme J Gregori J Diehl V et al. Improving perfusion quantification in arterial spin labeling for delayed arrival times by using optimized acquisition schemes. Z Med Phys 2015; 25: 221–229. 25125192 10.1016/j.zemedi.2014.07.003

[56] Martin SZ Madai VI von Samson-Himmelstjerna FC et al. 3D GRASE pulsed arterial spin labeling at multiple inflow times in patients with long arterial transit times: comparison with dynamic susceptibility-weighted contrast-enhanced MRI at 3 Tesla. J Cereb Blood Flow Metab 2015; 35: 392–401. 25407272 10.1038/jcbfm.2014.200 PMC4348376

[57] Lansberg MG Straka M Kemp S et al. MRI profile and response to endovascular reperfusion after stroke (DEFUSE 2): a prospective cohort study. Lancet Neurol 2012; 11: 860–867. 22954705 10.1016/S1474-4422(12)70203-X PMC4074206

[58] Calamante F Christensen S Desmond PM et al. The physiological significance of the time-to-maximum (Tmax) parameter in perfusion MRI. Stroke 2010; 41: 1169–1174. 20413735 10.1161/STROKEAHA.110.580670

[59] Christensen S Mouridsen K Wu O et al. Comparison of 10 perfusion MRI parameters in 97 sub-6-hour stroke patients using voxel-based receiver operating characteristics analysis. Stroke 2009; 40: 2055–2061. 19359626 10.1161/STROKEAHA.108.546069

[60] Gunther M. Encoded continuous arterial spin labeling. In: *ISMRM workshop on cerebral perfusion and brain function: Novel techniques and applications*, Salvador da Bahia, Brazil, 28 July–1 August 2007, abstract number: 3.

[61] Petersen ET Mouridsen K Golay X The QUASAR reproducibility study, Part II: Results from a multi-center Arterial Spin Labeling test-retest study. Neuroimage 2010; 49: 104–113. 19660557 10.1016/j.neuroimage.2009.07.068 PMC2768325

[62] Mutsaerts HJ, Petr J, Vaclavu L, et al. The spatial coefficient of variation in arterial spin labeling cerebral blood flow images. *J Cereb Blood Flow Metab*. Epub ahead of print January 2017. DOI: 10.1177/0271678X16683690. 10.1177/0271678X16683690 PMC5584689 28058975

[63] Grade M Hernandez Tamames JA Pizzini FB et al. A neuroradiologist's guide to arterial spin labeling MRI in clinical practice. Neuroradiology 2015; 57: 1181–1202. 26351201 10.1007/s00234-015-1571-z PMC4648972

[64] Teeuwisse WM Schmid S Ghariq E et al. Time-encoded pseudocontinuous arterial spin labeling: basic properties and timing strategies for human applications. Magn Reson Med 2014; 72: 1712–1722. 24395462 10.1002/mrm.25083

[65] von Samson-Himmelstjerna F Madai VI Sobesky J et al. Walsh-ordered hadamard time-encoded pseudocontinuous ASL (WH pCASL). Magn Reson Med 2016; 76: 1814–1824. 26714671 10.1002/mrm.26078

[66] von Samson-Himmelstjerna F, Chappell MA, Sobesky J, et al. Subtraction free arterial spin labeling: A new Bayesian-inference based approach for gaining perfusion data from time encoded data. In: *Proceedings of the international society for magentic resonance in medicine* (ISMRM), 2015, p.275.

[67] Wells JA Lythgoe MF Gadian DG et al. In vivo Hadamard encoded continuous arterial spin labeling (H-CASL). Magn Reson Med 2010; 63: 1111–1118. 20373414 10.1002/mrm.22266

[68] Dai W Shankaranarayanan A Alsop DC Volumetric measurement of perfusion and arterial transit delay using hadamard encoded continuous arterial spin labeling. Magn Reson Med 2013; 69: 1014–1022. 22618894 10.1002/mrm.24335 PMC3427721

[69] Liu P Uh J Lu H Determination of spin compartment in arterial spin labeling MRI. Magn Reson Med 2011; 65: 120–127. 20740655 10.1002/mrm.22601 PMC2994958

[70] Wells JA Siow B Lythgoe MF et al. Measuring biexponential transverse relaxation of the ASL signal at 9.4 T to estimate arterial oxygen saturation and the time of exchange of labeled blood water into cortical brain tissue. J Cereb Blood Flow Metab 2013; 33: 215–224. 23168531 10.1038/jcbfm.2012.156 PMC3564190

[71] Schmid S Teeuwisse WM Lu H et al. Time-efficient determination of spin compartments by time-encoded pCASL T2-relaxation-under-spin-tagging and its application in hemodynamic characterization of the cerebral border zones. Neuroimage 2015; 123: 72–79. 26297847 10.1016/j.neuroimage.2015.08.025

[72] Starr JM Farrall AJ Armitage P et al. Blood-brain barrier permeability in Alzheimer's disease: a case-control MRI study. Psychiatry Res 2009; 171: 232–241. 19211227 10.1016/j.pscychresns.2008.04.003

[73] Chen Z, Zhao X, Teeuwisse WM, et al. Incorporation of labeling efficiency measurement into a normal PCASL perfusion scan without SNR-penalty. In: *Proceedings of the international society for magnetic resonance in medicine* (ISMRM), Singapore, 2016, p.1009.

[74] Okell TW, Teeuwisse WM, Chappell MA, et al. Time-and vessel encoded PCASL: A free lunch with all the trimmings. In: *Proceedings of the international society for magnetic resonance imaging (ISMRM)*, Toronto, Canada, 30 May–5 June 2015, p.264.

[75] Hendrikse J Petersen ET van Laar PJ et al. Cerebral border zones between distal end branches of intracranial arteries: MR imaging. Radiology 2008; 246: 572–580. 18055872 10.1148/radiol.2461062100

[76] van Laar PJ Hendrikse J Golay X et al. In vivo flow territory mapping of major brain feeding arteries. Neuroimage 2006; 29: 136–144. 16095923 10.1016/j.neuroimage.2005.07.011

[77] Suzuki Y, Teeuwisse WM, Schmid S, et al. Simultaneous acquisition of perfusion maps and 4D MR angiography by means of arterial spin labeling MRI. In: *Proceedings of the international society for magnetic resonance in medicine (ISMRM)*, Milan, Italy, 10–16 May 2014, p.720.

[78] Winkelmann S Schaeffter T Koehler T et al. An optimal radial profile order based on the Golden Ratio for time-resolved MRI. IEEE Trans Med Imaging 2007; 26: 68–76. 17243585 10.1109/TMI.2006.885337

[79] Nehrke K Bornert P Prospective correction of affine motion for arbitrary MR sequences on a clinical scanner. Magn Reson Med 2005; 54: 1130–1138. 16200564 10.1002/mrm.20686

[80] Zun Z Shankaranarayanan A Zaharchuk G Pseudocontinuous arterial spin labeling with prospective motion correction (PCASL-PROMO). Magn Reson Med 2014; 72: 1049–1056. 24243585 10.1002/mrm.25024 PMC4048655

[81] Okell TW. Combined angiography and perfusion using radial imaging and arterial spin labeling. In: *Proceedings of the international society for magnetic resonance in medicine (ISMRM)*, Singapore, Singapore, 7 May–13 May 2016, p.1001.

[82] Barth M Breuer F Koopmans PJ et al. Simultaneous multislice (SMS) imaging techniques. Magn Reson Med 2016; 75: 63–81. 26308571 10.1002/mrm.25897 PMC4915494

[83] Kim T Shin W Zhao T et al. Whole brain perfusion measurements using arterial spin labeling with multiband acquisition. Magn Reson Med 2013; 70: 1653–1661. 23878098 10.1002/mrm.24880

[84] Li X Wang D Auerbach EJ et al. Theoretical and experimental evaluation of multi-band EPI for high-resolution whole brain pCASL Imaging. Neuroimage 2015; 106: 170–181. 25462690 10.1016/j.neuroimage.2014.10.029 PMC4337884

[85] Feinberg DA Beckett A Chen L Arterial spin labeling with simultaneous multi-slice echo planar imaging. Magn Reson Med 2013; 70: 1500–1506. 24130105 10.1002/mrm.24994 PMC4162886

[86] Wang Y Moeller S Li X et al. Simultaneous multi-slice Turbo-FLASH imaging with CAIPIRINHA for whole brain distortion-free pseudo-continuous arterial spin labeling at 3 and 7 T. Neuroimage 2015; 113: 279–288. 25837601 10.1016/j.neuroimage.2015.03.060 PMC4433786

[87] Ma D Gulani V Seiberlich N et al. Magnetic resonance fingerprinting. Nature 2013; 495: 187–192. 23486058 10.1038/nature11971 PMC3602925

[88] Su P, Mao D, Liu P, et al. Arterial spin labeling without control/label pairing and post-labeling delay: An MR fingerprinting implementation. In: *Proceedings of the international society for magnetic resonance in medicine (ISMRM)*, Singapore, Singapore, 7–13 May 2016, p.276.

[89] Wright KL, Ma D, Jiang Y, et al. Theoretical framework for MR fingerprinting with ASL: Simultaneous quantification of CBF, transit time, and T1. In: *Proceedings of the international society for magnetic resonance in medicine (ISMRM)*, Milan, Italy, 10–16 May 2014, p.417.

[90] Taei-Tehrani MR van Osch MJ Brown TR Pseudo-random arterial modulation (PRAM): a novel arterial spin labeling approach to measure flow and blood transit times. J Magn Reson Imaging 2012; 35: 223–228. 21990142 10.1002/jmri.22844

[91] Lustig M Donoho D Pauly JM Sparse MRI: The application of compressed sensing for rapid MR imaging. Magn Reson Med 2007; 58: 1182–1195. 17969013 10.1002/mrm.21391

[92] Han PK Ye JC Kim EY et al. Whole-brain perfusion imaging with balanced steady-state free precession arterial spin labeling. NMR Biomed 2016; 29: 264–274. 26676386 10.1002/nbm.3463

[93] Zhao L Fielden SW Feng X et al. Rapid 3D dynamic arterial spin labeling with a sparse model-based image reconstruction. Neuroimage 2015; 121: 205–216. 26169322 10.1016/j.neuroimage.2015.07.018 PMC4615585

[94] Wong EC Cronin M Wu WC et al. Velocity-selective arterial spin labeling. Magn Reson Med 2006; 55: 1334–1341. 16700025 10.1002/mrm.20906

[95] Schmid S Heijtel DF Mutsaerts HJ et al. Comparison of velocity- and acceleration-selective arterial spin labeling with [15O]H_2_O positron emission tomography. J Cereb Blood Flow Metab 2015; 35: 1296–1303. 25785831 10.1038/jcbfm.2015.42 PMC4528003

[96] Wells JA, Thomas DL, Saga T, et al. MRI of cerebral micro-vascular flow patterns: A multi-direction diffusion-weighted ASL approach. *J Cereb Blood Flow Metab* 2017; 37: 2076–2083. 10.1177/0271678X16660985 PMC5464702 27461904

[97] Silva AC Williams DS Koretsky AP Evidence for the exchange of arterial spin-labeled water with tissue water in rat brain from diffusion-sensitized measurements of perfusion. Magn Reson Med 1997; 38: 232–237. 9256102 10.1002/mrm.1910380211

[98] Guo J Wong EC Increased SNR efficiency in velocity selective arterial spin labeling using multiple velocity selective saturation modules (mm-VSASL). Magn Reson Med 2015; 74: 694–705. 25251933 10.1002/mrm.25462 PMC4369468

[99] Wu WC Wong EC Feasibility of velocity selective arterial spin labeling in functional MRI. J Cereb Blood Flow Metab 2007; 27: 831–838. 16926843 10.1038/sj.jcbfm.9600386

[100] Wu WC Wong EC Intravascular effect in velocity-selective arterial spin labeling: the choice of inflow time and cutoff velocity. Neuroimage 2006; 32: 122–128. 16713716 10.1016/j.neuroimage.2006.03.001

[101] Wong EC Buxton RB Frank LR Implementation of quantitative perfusion imaging techniques for functional brain mapping using pulsed arterial spin labeling. NMR Biomed 1997; 10: 237–249. 9430354 10.1002/(sici)1099-1492(199706/08)10:4/5<237::aid-nbm475>3.0.co;2-x

[102] Frank LR Lu K Wong EC Perfusion tensor imaging. Magn Reson Med 2008; 60: 1284–1291. 19030161 10.1002/mrm.21806 PMC4319714

[103] Luo T and Hernandez-Garcia L. Inflow velocity density mapping using Fourier analysis of velocity selective ASL images. In: *Proceedings of the international society for magnetic resonance in medicine (ISMRM)*, Toronto, Canada, 30 May–5 June 2015, p.2334.

[104] Ostergaard L Aamand R Gutierrez-Jimenez E et al. The capillary dysfunction hypothesis of Alzheimer's disease. Neurobiol Aging 2013; 34: 1018–1031. 23084084 10.1016/j.neurobiolaging.2012.09.011

[105] Jespersen SN Ostergaard L The roles of cerebral blood flow, capillary transit time heterogeneity, and oxygen tension in brain oxygenation and metabolism. J Cereb Blood Flow Metab 2012; 32: 264–277. 22044867 10.1038/jcbfm.2011.153 PMC3272609

[106] Hendrikse J Van der Grond J Lu H et al. Flow territory mapping of the cerebral arteries with regional perfusion MRI. Stroke 2004; 35: 882–887. 14988567 10.1161/01.STR.0000120312.26163.EC

[107] van Laar PJ Hendrikse J Golay X et al. In vivo flow territory mapping of major brain feeding arteries. Neuroimage 2006; 29: 136–144. 16095923 10.1016/j.neuroimage.2005.07.011

[108] Wong EC Vessel-encoded arterial spin-labeling using pseudocontinuous tagging. Magn Reson Med 2007; 58: 1086–1091. 17969084 10.1002/mrm.21293

[109] Gevers S Bokkers RP Hendrikse J et al. Robustness and reproducibility of flow territories defined by planning-free vessel-encoded pseudocontinuous arterial spin-labeling. AJNR Am J Neuroradiol 2012; 33: E21–E25. 21393410 10.3174/ajnr.A2410 PMC7964817

[110] Wong EC Guo J Blind detection of vascular sources and territories using random vessel encoded arterial spin labeling. MAGMA 2012; 25: 95–101. 22231782 10.1007/s10334-011-0302-7

[111] Helle M Norris DG Rufer S et al. Superselective pseudocontinuous arterial spin labeling. Magn Reson Med 2010; 64: 777–786. 20597127 10.1002/mrm.22451

[112] Dai W Robson PM Shankaranarayanan A et al. Modified pulsed continuous arterial spin labeling for labeling of a single artery. Magn Reson Med 2010; 64: 975–982. 20665896 10.1002/mrm.22363 PMC3266713

[113] Helle M Rufer S van Osch MJ et al. Superselective arterial spin labeling applied for flow territory mapping in various cerebrovascular diseases. J Magn Reson Imaging 2013; 38: 496–503. 23526786 10.1002/jmri.24041

